# Comprehending toll-like receptors: pivotal element in the pathogenesis of sepsis and its complications

**DOI:** 10.3389/fimmu.2025.1591011

**Published:** 2025-05-02

**Authors:** Wei Wang, Shengtian Mu, Dongli Yan, Huan Qin, Zhen Zheng

**Affiliations:** Department of Intensive Care Unit, Cancer Hospital of China Medical University, Liaoning Cancer Hospital and Institute, Shenyang, Liaoning, China

**Keywords:** sepsis, sepsis associated complications, toll-like receptors (TLRs), damage-associated molecular patterns (DAMPs), pathogen-associated molecular patterns (PAMPs), inflammation

## Abstract

Sepsis, a critical systemic inflammatory response syndrome elicited by pathogenic microorganisms, poses a significant challenge in clinical practice due to its rapid progression and potential for multi-organ failure. This review delineates the intricate roles of Toll-like receptors (TLRs), essential components of the innate immune system, in mediating host responses during sepsis. TLRs recognize pathogen-associated molecular patterns (PAMPs) and damage-associated molecular patterns (DAMPs), thereby initiating signaling cascades that lead to the synthesis of pro-inflammatory cytokines and chemokines. However, the dysregulation of TLR signaling can precipitate a hyper-inflammatory state known as a “cytokine storm,” characterized by excessive tissue damage and complications such as Acute Respiratory Distress Syndrome (ARDS) and acute kidney injury (AKI). Several therapeutic strategies targeting TLR pathways are under exploration to mitigate the adverse effects of sepsis. Despite advancements, significant gaps remain, including the need for robust clinical validation and understanding of TLR expression variability among individuals. Future research should focus on elucidating the precise molecular mechanisms governing TLR-mediated responses and developing human-specific therapeutic interventions. This review aims to consolidate current knowledge on TLRs in sepsis, highlighting their dual roles as both defenders against infection and contributors to pathological conditions, thereby informing future therapeutic strategies.

## 1 Introduction

Sepsis is a life-threatening condition caused by an uncontrolled immune response to infection, leading to high morbidity and mortality. It features immune dysfunction, with an initial exaggerated inflammatory response followed by sustained immunosuppression (1). Early on, immune cells like monocytes and macrophages release inflammatory mediators and recruit neutrophil extracellular traps (NETs) to fight pathogens. However, excessive inflammation can cause immune cell death, releasing damage-associated molecular patterns (DAMPs) that lead to endothelial dysfunction, disseminated intravascular coagulation (DIC), and organ failure. This immune cell depletion results in persistent immunosuppression, increasing the risk of secondary infections, especially from multidrug-resistant organisms, which are a major cause of death in later stages (2, 3). Common sources of sepsis include pneumonia, urinary tract infections, abdominal infections, bloodstream infections, skin infections, and viral infections. Treatment involves early fluid resuscitation, source control, antibiotics, vasoactive agents, glycemic control, nutrition support, corticosteroids, blood products, immunomodulation, blood purification, anticoagulation, renal replacement therapy, and mechanical ventilation (4). The pathogenesis of sepsis is complex, involving systemic inflammation, immune dysregulation, and organ dysfunction. Molecular mechanisms include dysregulated inflammation, immunosuppression, coagulation abnormalities, immune cell apoptosis, and endoplasmic reticulum stress. However, the exact molecular mechanisms of sepsis are still unclear, highlighting the need for effective monitoring and treatment strategies (5).

Toll-like receptors (TLRs) are innate immune receptors that primarily include type I transmembrane and pathogen pattern recognition receptors. They recognize PAMPs and are essential for acute inflammatory responses, apoptosis, and signal transduction. There are 10 TLRs in humans and 12 in mice. TLRs consist of three domains: extracellular, transmembrane, and intracellular (6, 7). They are divided into two groups based on localization and ligands: cell surface TLRs (TLR1, TLR2, TLR4, TLR5, TLR6, TLR11) that recognize microbial membrane components, and intracellular TLRs (TLR3, TLR7, TLR8, TLR9) that recognize microbial nucleic acids. TLRs are expressed on immune cells (e.g., DCs, macrophages, lymphocytes) and non-immune cells (e.g., epithelial, endothelial cells) (8). They bind PAMPs, initiating signaling cascades that release inflammatory mediators. Recognition of bacterial PAMPs by TLRs (TLR2, TLR4, TLR5, TLR9) activates NF-κB via MyD88-dependent and -independent pathways, leading to pro-inflammatory cytokine production and potentially sepsis (9, 10). This article critically reviewed the recent investigations on the role of TLRs signaling in sepsis and sepsis-associated complications, as well as the clinical trials that tested the therapeutic efficacy of blocking TLRs.

## 2 The overview of TLRs

Toll-like receptors (TLRs) are essential components of the innate immune system known for their distinctive structure, comprising three main domains: extracellular, transmembrane, and cytoplasmic. The extracellular domain of TLRs plays a critical role in initiating the body’s response to external threats and tissue damage, while the transmembrane domain regulates their subcellular location (11). The cytoplasmic Toll/interleukin-1 receptor (TIR) domain acts as a docking site for downstream adapters in signaling cascades, leading to the production of inflammatory mediators and activation of immune cells. TLRs interact with both external pathogens and endogenous molecules, influencing immune responses and inflammation. Disruptions in TLR function are linked to various immune-related disorders, underscoring the importance of understanding TLR activity in immune-mediated diseases and the development of therapeutic interventions (12).

**Figure 1:**
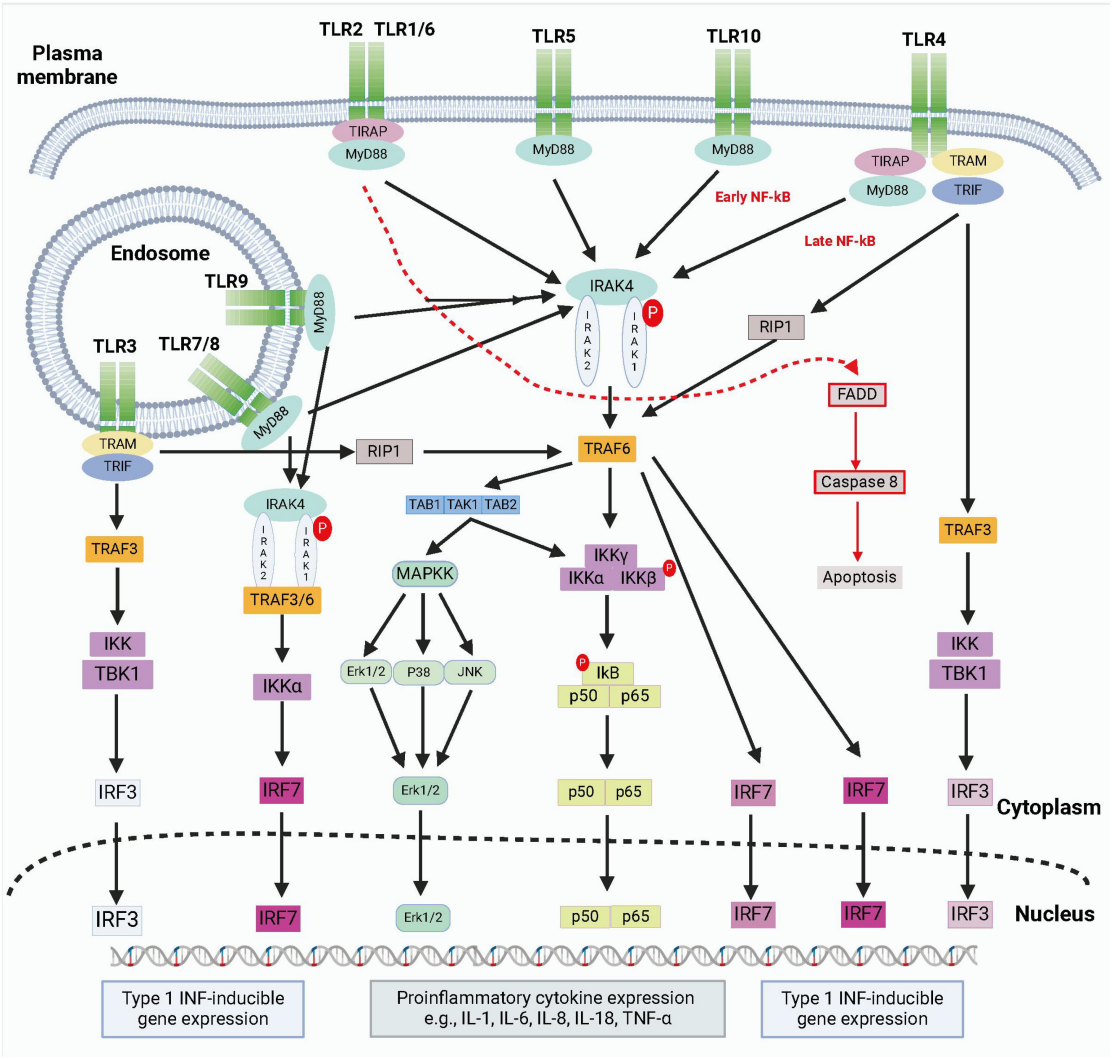
The brief summary of TLR signaling pathways. TLRs detect PAMPs or DAMPs on plasma membrane or endosomal membranes. TLRs activate two main pathways: the MyD88-dependent and MyD88-independent pathways. The MyD88-dependent pathway involves MyD88 recruitment and myddosome formation, leading to NF-κB release and proinflammatory cytokine transcription via IKKγ complex activation and MAPK stimulation. The myddosome also promotes IRF5 and IRF7 production, inducing type I IFN gene expression. The MyD88-independent pathway is activated by TLR3 and TLR4, recruiting TRIF to form a complex with TRAF3, TBK1, and IKK. This complex promotes IRF3 nuclear translocation and late-phase NF-κB activation. Both pathways stimulate type I IFN production. Created by biorender.com.

TLRs are divided into two groups based on their location and the types of pathogen patterns they recognize: cell surface TLRs and endosomal TLRs. Cell surface TLRs, such as TLR1, TLR2, TLR4, and TLR6, detect extracellular pathogen patterns, while endosomal TLRs like TLR3, TLR7, and TLR9 identify internalized viral or bacterial DNA/RNA (13). Each TLR subtype plays a distinct role in immune responses by activating downstream signaling molecules and initiating specific immune reactions. Imbalances in TLR signaling pathways can lead to chronic inflammatory conditions, autoimmune diseases, or immunodeficiency disorders, emphasizing the critical role of TLRs in mounting responses to various pathogens (**Figure 1**).

Activation of TLRs involves interactions with pathogen-associated molecular patterns (PAMPs), triggering downstream signaling pathways that lead to the expression of pro-inflammatory molecules and immune-regulatory factors. While TLRs play a pivotal role in combating infections, excessive TLR activation can result in chronic inflammation or autoimmune diseases (14, 15). Strict regulatory mechanisms, including negative feedback loops and regulatory cells, help maintain immune balance and prevent hyperactivation of TLR signaling. Understanding the mechanisms governing TLR activation and regulation is crucial for developing novel therapeutic strategies and targeting TLRs to treat immune-related diseases effectively.

## 3 The roles of TLRs in sepsis and sepsis-associated complications

**Figure 2:**
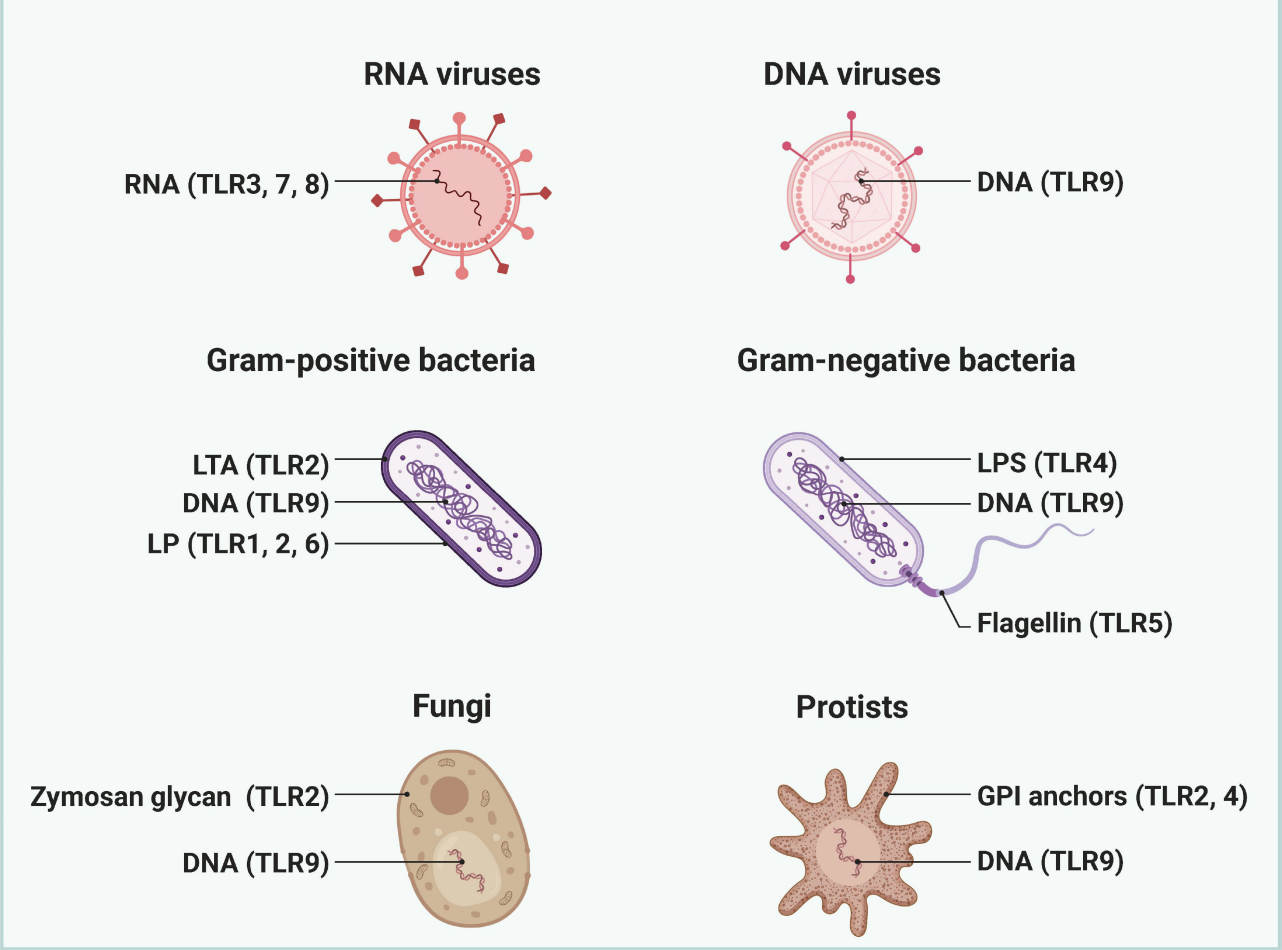
The pathogen ligand targets for different TLRs. Different TLRs are widely recognized as key receptors for recognizing and responding to specific pathogen ligands. Created by biorender.com.

Toll-like receptors (TLRs) are integral components of the innate immune system, playing a pivotal role in the recognition of pathogens and the initiation of immune responses (**Figure 2**). Their involvement in sepsis—a life-threatening condition characterized by a dysregulated host response to infection—has garnered significant attention in recent years. This section explores the multifaceted roles of TLRs in sepsis and the complications that arise from their activation (16). TLRs are a family of pattern recognition receptors (PRRs) that detect pathogen-associated molecular patterns (PAMPs) and damage-associated molecular patterns (DAMPs). In humans, there are ten known TLRs (TLR1-10), each with specific ligands, including bacterial lipopolysaccharides (LPS), peptidoglycans, and viral nucleic acids (17). TLRs are primarily expressed on immune cells such as macrophages, dendritic cells, and neutrophils, but they are also found on non-immune cells, indicating their broad role in immune regulation. In sepsis, TLRs are crucial for the early detection of invading pathogens (18). Upon recognition of PAMPs, TLRs activate intracellular signaling pathways, primarily through the MyD88-dependent and MyD88-independent pathways. This activation leads to the production of pro-inflammatory cytokines, chemokines, and other mediators that orchestrate the immune response. However, the activation of TLRs in sepsis can become dysregulated. While initial TLR activation is essential for pathogen clearance, excessive or prolonged TLR signaling can result in a hyper-inflammatory state, contributing to tissue damage and organ dysfunction. This phenomenon is often referred to as a “cytokine storm,” where an overwhelming release of inflammatory mediators exacerbates the condition, leading to complications such as acute respiratory distress syndrome (ARDS), acute kidney injury, and multiple organ failure (19, 20).

### 3.1 Sepsis-associated complications

**Figure 3:**
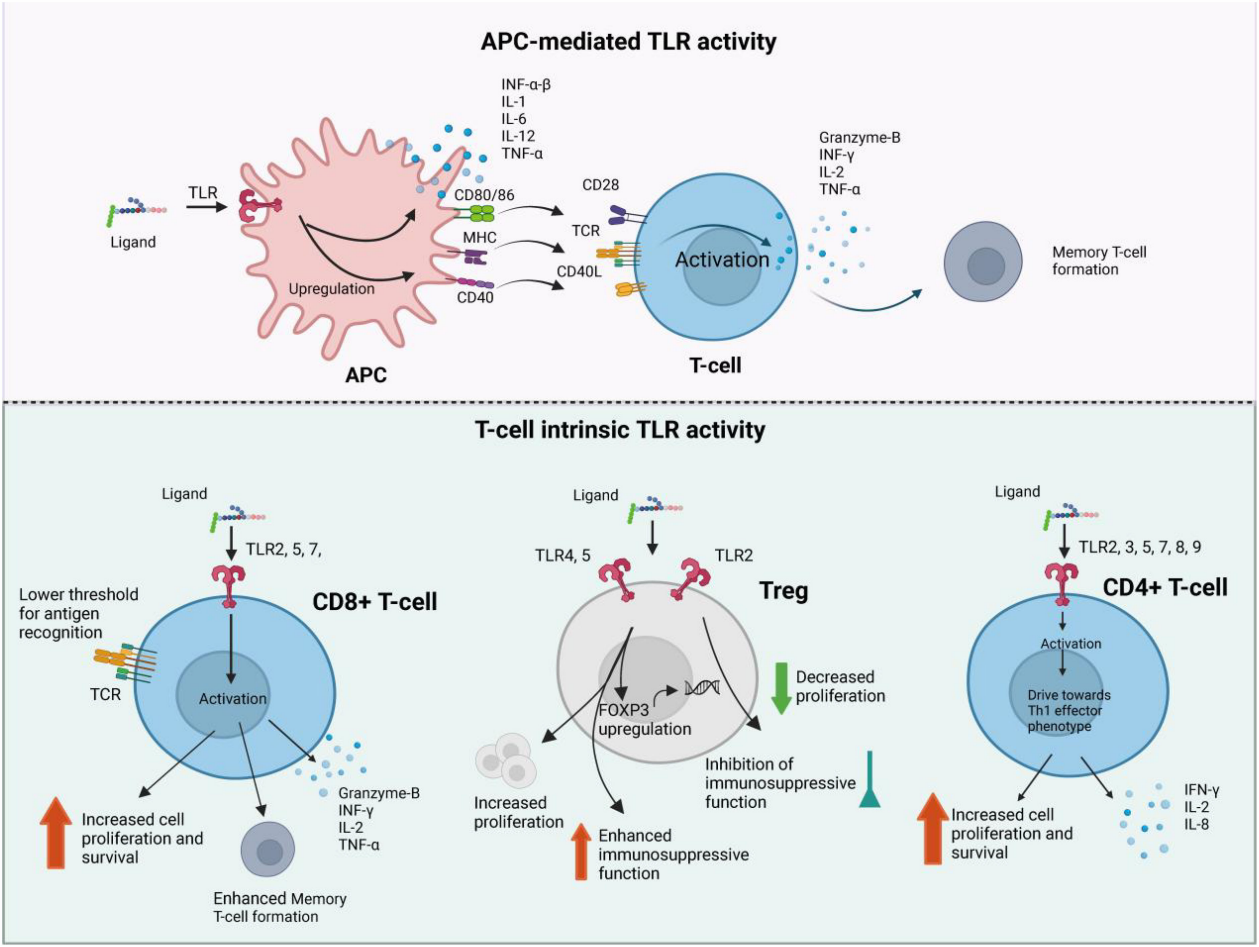
The brief pathogenesis of sepsis. Sepsis pathogenesis entails proinflammatory and anti-inflammatory mechanisms. Proinflammatory and anti-inflammatory responses coexist, but sepsis disrupts their dynamic equilibrium. Initially, there is massive proinflammatory factor secretion, immune cell reprogramming, complement and coagulation system activation, leading to a cytokine storm. Anti-inflammatory cytokines may be released subsequently or concurrently. As sepsis progresses, immune system over-activation results in immune cell depletion and immunosuppression. Sepsis-induced immunosuppression involves immune cell regulation, apoptosis autophagy, endotoxin tolerance, central nervous system changes, and metabolic alterations. Targeting immunosuppression in sepsis can be achieved through these mechanisms. Antigen-presenting cells (APCs), including dendritic cells and macrophages, secrete cytokines such as interferon-α/β (IFN-α/β), interleukin-1 (IL-1), IL-2, and tumor necrosis factor-α (TNF-α). These cells express surface markers CD80/86, MHC class II, and CD40, which facilitate interaction with T-cell receptors (TCR) and co-stimulatory molecule CD28, leading to activation of naïve T-cells. CD8+ cytotoxic T-cells release IFN-γ, IL-2, and TNF-α, alongside the apoptosis-inducing effector molecule Granzyme-B, enhancing their proliferation and cytotoxic activity. CD8+ cytotoxic T-cells release IFN-γ, IL-2, and TNF-α, alongside the apoptosis-inducing effector molecule Granzyme-B, enhancing their proliferation and cytotoxic activity.

The dysregulation of TLR signaling in sepsis can lead to various complications: Acute Respiratory Distress Syndrome (ARDS): TLR activation contributes to the inflammatory response in the lungs, leading to increased vascular permeability, pulmonary edema, and impaired gas exchange. ARDS is a common and severe complication of sepsis, often requiring mechanical ventilation. Acute Kidney Injury (AKI): TLRs are implicated in renal inflammation and injury during sepsis. The activation of TLRs in renal cells can lead to the release of inflammatory mediators, resulting in tubular damage and impaired renal function (21). Multiple Organ Dysfunction Syndrome (MODS): The systemic inflammatory response triggered by TLR activation can affect multiple organ systems, leading to MODS. This condition is characterized by the failure of two or more organ systems and is a leading cause of death in sepsis patients (**Figure 3**).

### 3.2 TLRs in sepsis-associated kidney injury

Research indicates a close association between the TLR family and the onset and progression of septic shock accompanied by AKI. TLRs play a pivotal role in recognizing PAMPs present in the cell walls of pathogenic microorganisms. Upon recognition, TLRs trigger nonspecific immune responses, leading to the release of numerous inflammatory mediators. Excessive production of exogenous and endogenous inflammatory mediators and pathogens can result in a rapid increase in TLR levels, ultimately causing damage to renal tubular cells (22, 23). Therefore, timely and effective clearance of these inflammatory mediators and pathogens, along with reducing TLR levels, is crucial for protecting renal function, lowering blood urea nitrogen, uric acid, and creatinine levels, and maintaining stable blood circulation in septic shock with AKI. Understanding the intricate mechanisms underlying TLRs in sepsis-associated kidney injury is imperative for the prevention and treatment of these complications. Future research endeavors should delve deeper into the regulatory mechanisms of TLRs to unveil new avenues and strategies for intervening in sepsis-associated kidney injury (24).

Jia and colleagues have unveiled a significant finding regarding AKI, demonstrating that targeted ablation of the chemokine CCL2 specifically within the proximal renal tubules can mitigate kidney damage by reducing macrophage infiltration and the expression of pro-inflammatory cytokines. Their study suggests that endogenous release of CCL2 within proximal renal tubules might contribute to renal inflammation and AKI induced by sepsis (25, 26). Moreover, they identified that TLR2 signaling in tubular epithelial cells (TECs) activates NF-κB, consequently promoting the upregulation of CCL2. These results propose that targeting the tubular TLR2/NF-κB/CCL2 signaling pathway holds promise as a potential therapeutic strategy for addressing purulent AKI. Furthermore, Senousy et al. conducted research on α-antitrypsin (α-AT), revealing its anti-inflammatory and antioxidant properties and evaluating its impact on lung, liver, and kidney damage in sepsis. Their experimental findings demonstrated that α-AT treatment improved the survival rate of septic rats while reducing levels of oxidative stress markers and inflammatory cytokines. The protective effects of α-AT were further corroborated through histopathological examinations of organ tissues. This study suggests that α-AT exerts its beneficial effects by inhibiting the TLR4/NF-κB pathway, thus mitigating oxidative stress and inflammatory responses (27).

The identification of the tubular TLR2/NF-κB/CCL2 signaling pathway and the inhibition of the TLR4/NF-κB pathway represent promising strategies for addressing purulent AKI and related renal injuries in sepsis. However, further research is imperative to validate these mechanisms and ascertain their feasibility and efficacy in clinical treatment (28).

### 3.3 TLRs in sepsis-associated liver injury

In sepsis-associated liver injury, TLRs assume a pivotal role. The liver, being the body’s primary immunoregulatory organ, harbors a diverse array of immune cells and molecules, with TLRs serving as central mediators of immune responses. During sepsis, pathogens or their constituents infiltrate the liver through the bloodstream, activating hepatic TLRs and instigating a cascade of immune reactions. While these responses aid in pathogen clearance, they can also precipitate liver tissue damage. TLRs discern specific pathogen components, such as bacterial LPS recognized by TLR4, prompting liver macrophages and Kupffer cells to secrete inflammatory cytokines like TNF-α, IL-6, and IL-1β. While these cytokines assist in controlling infections, an exaggerated response can result in hepatocyte injury (29, 30). Furthermore, TLR activation can induce hepatocyte apoptosis, particularly in severe sepsis patients, by activating intrinsic and extrinsic apoptosis pathways, thus exacerbating sepsis severity. Future research should delve deeper into the precise mechanisms of TLRs in liver injury and explore how these mechanisms can be leveraged to devise novel therapeutic strategies. Shalmani et al., through sepsis induction in rats and subsequent treatment with Monomethyl fumarate (MMF), dexamethasone, or a carrier, observed significant reductions in inflammatory markers and liver enzyme levels in septic rats (31). Moreover, MMF potentially attenuates sepsis-induced liver dysfunction by inhibiting the TLR-4/NF-κB signaling pathway (32). This suggests that MMF could ameliorate liver function in sepsis patients by modulating immune and antioxidant responses, thereby presenting a novel therapeutic avenue for sepsis treatment.

### 3.4 TLRs in sepsis-associated intestinal injury

In sepsis, intestinal damage plays a pivotal role in disease pathogenesis and progression. Sepsis-associated intestinal injury encompasses pathological alterations in the intestines induced by sepsis, frequently observed in critically ill patients and severe infection scenarios. It constitutes a complex pathological process involving multifaceted mechanisms and interactions among various cell types. In sepsis-associated intestinal injury, inflammation, bacterial translocation, alterations in intestinal mucosal blood flow, and heightened intestinal permeability are prominent pathophysiological features. These changes not only impair intestinal function but also render the intestine a reservoir of toxins and exogenous microbial components, thereby exacerbating inflammation and sepsis development (33).

In studies investigating sepsis-associated intestinal injury, multiple research teams have explored the role and mechanisms of TLRs. Li et al. observed upregulation of Pentraxin 3 (PTX3) in a sepsis model, which exerts anti-inflammatory effects by inhibiting the TLR signaling pathway. Their findings suggest that PTX3 regulates TLRs signaling and protects against sepsis-induced intestinal mucosal damage (34). Additionally, Panpetch et al. demonstrated that the combined action of Candida albicans and Klebsiella pneumoniae amplifies inflammation in intestinal and liver cells, as well as macrophages, disrupting the intestinal flora balance and inducing excessive inflammatory responses (35). This study underscores the role of Candida albicans in exacerbating inflammation and impairing intestinal barrier function, thus enhancing our understanding of sepsis-associated intestinal injury mechanisms (36). Furthermore, Shen et al. showed that inhibiting C5a receptor 1 (C5ar1) reduces inflammatory markers, mitigates tissue damage, and suppresses NET formation, thereby ameliorating organ damage in septic rats. These findings further underscore the significant involvement of the TLRs signaling pathway in sepsis-associated intestinal injury (37). Overall, these studies deepen our comprehension of TLRs’ role and mechanisms in sepsis-associated intestinal injury. Further research can elucidate the specific regulatory mechanisms of the TLRs signaling pathway in this pathological process, offering new treatment strategies and targets. However, the feasibility and safety of these approaches require further validation. Future research should delve into the interactions between the TLRs signaling pathway and other cells and factors, along with its comprehensive regulatory mechanism in sepsis-associated intestinal injury (38, 39).

### 3.5 TLRs in sepsis-associated heart injury

In sepsis-associated heart injury, Toll-like receptors (TLRs) play a significant role in initiating and perpetuating the inflammatory response that contributes to cardiac dysfunction. TLR2 and TLR4 are the primary TLRs implicated in sepsis-related cardiac injury. During sepsis, TLR2 and TLR4 recognize pathogen-associated molecular patterns (PAMPs) derived from invading microorganisms, such as lipopolysaccharide (LPS) from Gram-negative bacteria (40, 41). Activation of TLR2 and TLR4 leads to the recruitment of adaptor molecules, including myeloid differentiation primary response 88 (MyD88), which initiates downstream signaling pathways. The primary signaling pathway activated by TLR2 and TLR4 in sepsis is the nuclear factor-kappa B (NF-κB) pathway. NF-κB is a transcription factor that controls the expression of pro-inflammatory cytokines, such as tumor necrosis factor-alpha (TNF-α), interleukin-1 beta (IL-1β), and interleukin-6 (IL-6) (42, 43). These cytokines contribute to the systemic inflammatory response observed in sepsis. TLR activation also induces the production of other inflammatory mediators, including chemokines and adhesion molecules, which promote the recruitment and activation of immune cells, such as neutrophils and macrophages, within the myocardium. This immune cell infiltration further amplifies the inflammatory response and contributes to tissue damage. In addition to the NF-κB pathway, TLR signaling in sepsis-associated cardiac injury can activate other pathways, including mitogen-activated protein kinases (MAPKs) pathways, such as the p38 MAPK and c-Jun N-terminal kinase (JNK) pathways. These pathways regulate the production of inflammatory cytokines and contribute to the development of cardiac dysfunction. The pro-inflammatory response elicited by TLR activation in sepsis-associated cardiac injury can lead to several detrimental effects (44, 45).

Tumor Necrosis Factor-alpha (TNF-alpha) has been identified as the principal cytokine induced by Toll-like receptor 4 (TLR4) in the pathophysiology of myocardial ischemia-reperfusion (I/R) injury (46, 47). Extensive animal studies conducted from 1986 to 1998 have established that TNF-alpha acts as a significant cardiodepressant, impairing myocardial contractility. Corresponding clinical evidence further corroborates the role of TNF-alpha in engendering myocardial dysfunction and hemodynamic perturbations subsequent to cardiopulmonary bypass (CPB) procedures. The acute negative inotropic effects of TNF-alpha are manifested through mechanisms that disrupt calcium homeostasis, induce cytotoxicity, impair excitation-contraction coupling, desensitize beta- and alpha-adrenergic catecholamine receptors, and promote the secretion of other myocardial depressants such as interleukin-1 beta (IL-1beta) (48). There are two distinct temporal mechanisms through which TNF-alpha exerts these inotropic effects: an early and a delayed phase. In the initial inflammatory response, TNF-alpha has been shown to depress myocyte adrenergic responsiveness independently of nitric oxide (NO), potentially mediated by alterations in sphingolipid metabolism and intracellular calcium transients (49). Conversely, during the later stages, TNF-alpha induces cardiac dysfunction via an NO-dependent mechanism, involving the synthesis of NO and resulting in reduced myofilament sensitivity to calcium (50). Another pivotal role of TNF-alpha in cardiac depression is its induction of myocardial apoptosis—a programmed cell death pathway which preserves cell membrane integrity and does not elicit creatine kinase release (51). Apoptotic cardiac cells may maintain contractility in response to calcium ionophores despite the onset of apoptosis. Myocardial apoptosis is primarily triggered through TNF-alpha receptor-1 and TNF-alpha receptor-2, both of which contain a death domain (TRADD). Signal transduction pathways activated by various stimuli propagate apoptotic signals that lead to the activation of caspases, culminating in DNA fragmentation and cellular demise (52). This apoptotic process is speculated to be mediated by sphingosine and NO. Clinically, cardiac myocyte apoptosis has been associated with an array of pathologies, including chronic heart failure, arrhythmogenic right ventricular dysplasia, viral myocarditis, dilated cardiomyopathy, and cardiogenic arrest. The therapeutic potential of TNF-alpha antagonists has been demonstrated in their capacity to mitigate cardiac infarct size in myocardial I/R injuries and enhance cardiac function, predominantly.

## 4 Diagnostic significance of TLRs in sepsis and associated complications

Research has underscored the pivotal role of TLRs in the diagnosis and management of sepsis and its associated complications. Initially, Larkin et al. observed thrombocytopenia in septic shock patients, correlating it with TLR-4 expression and platelet consumption, thus shedding light on the pathophysiological mechanism underlying septic shock (53). Concurrently, Williams et al. investigated TLR signaling in mouse sepsis, highlighting the significance of TLR2 and TLR7 in modulating coagulation function, platelet count, and tissue factor expression. Moreover, Sohn et al. elucidated the link between COVID-19 and TLRs, revealing upregulation of TLR4-mediated inflammatory signaling molecules and identifying S100A9 as a potential biomarker, offering insights into targeting TLR4-mediated inflammation therapeutically (54). Conversely, Bick et al. reported immunosuppression in sepsis patients, implying a limited response to immune checkpoint inhibitors, underscoring the importance of considering individual immune status and inflammatory responses in personalized sepsis treatment plans. Further investigations have expanded our understanding of TLR involvement in sepsis. For instance, Zhang et al. demonstrated correlations between TLR4 and TIRAP protein expression levels and sepsis severity, emphasizing their role in pathogenesis (55). Similarly, Álvarez-Estrada et al. identified increased TLR2, Siglec-3, and CD163 expression in a porcine sepsis model, indicating monocyte regulation in sepsis response. Additionally, ATALAN et al. noted lower serum TLR-9 levels in sepsis patients, with elevated levels associated with lactate levels above 5 mmol/l, suggesting a potential role in sepsis-induced immune suppression. Thon et al. highlighted TLR4 as the key LPS receptor activating immune responses, with significantly higher activation rates observed in sepsis patients compared to healthy individuals, offering prospects for targeted therapy based on TLR-4 activation status (56). Buys et al. revealed heterogeneous ex vivo cytokine production responses to TLR blockade in sepsis patients, emphasizing the importance of tailored ex vivo testing before immunomodulatory therapy initiation. Despite these advancements, the heterogeneity and complexity of study findings necessitate further research to deepen our understanding of TLR mechanisms in sepsis and enhance diagnostic and therapeutic strategies (57).

## 5 Discussion and prospects

**Figure 4:**
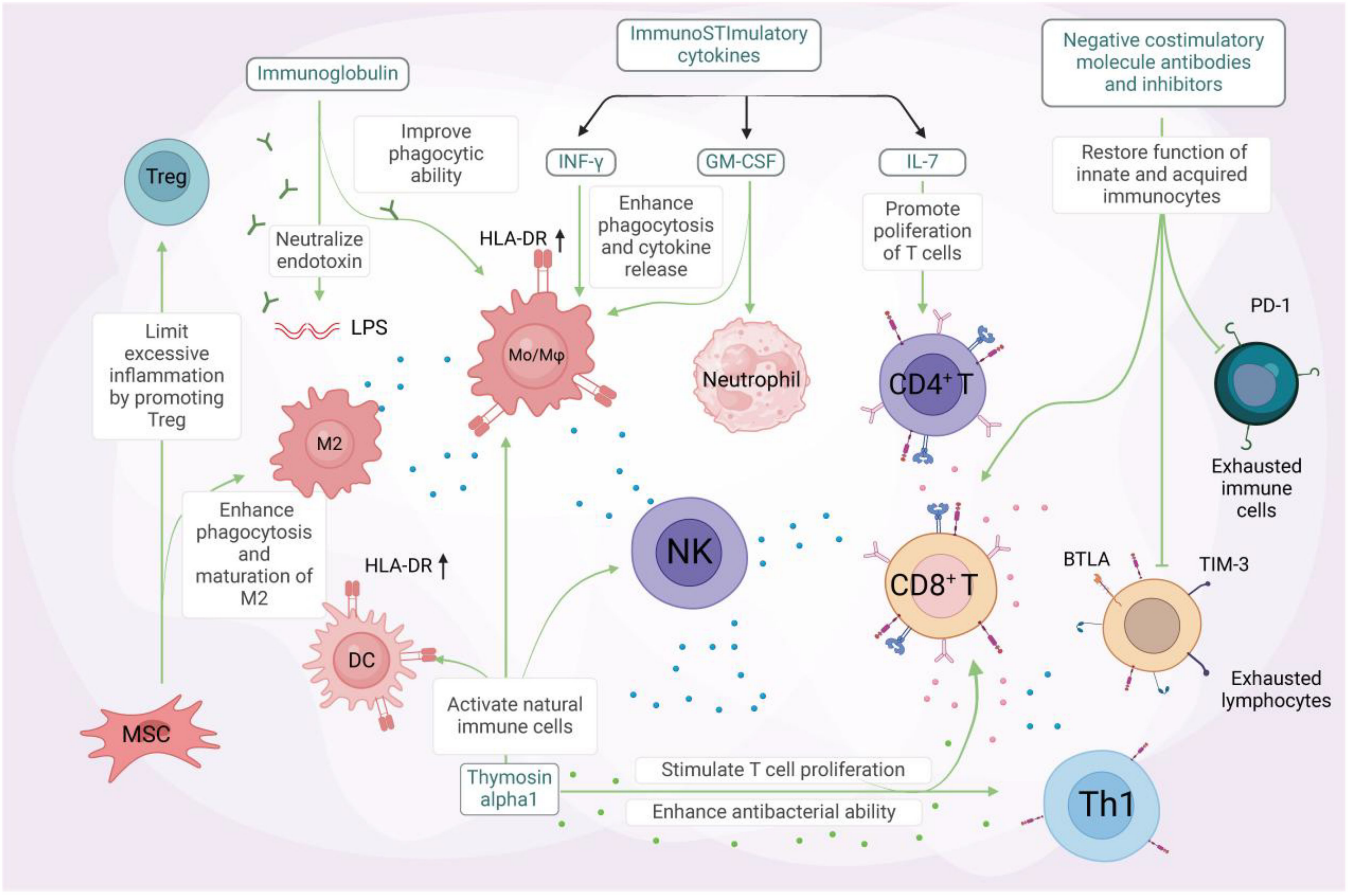
Unlocking the potential of immunotherapy in sepsis treatment. Immunomodulatory therapy, combined with anti-inflammatory measures, has been shown to effectively improve the outcome of severe sepsis. Certain immunostimulatory cytokines, including IFN-γ, GM-CSF, and IL-7, have been demonstrated to activate immune cells in sepsis. IFN-γ and GM-CSF enhance phagocytosis and pro-inflammatory cytokine release, as well as the expression of mHLA-DR on antigen-presenting cells (APCs). GM-CSF (middle panel) stimulates neutrophil differentiation and activation through JAK2/STAT5 signaling, while also promoting the survival and proliferation of other myeloid cells (e.g., macrophages and dendritic cells). IL-7 (right panel) drives CD4+ T cell development and homeostasis via JAK1/STAT5 activation, with additional effects on lymphoid lineage cells (including CD8+ T cells and B cell precursors). IL-7, on the other hand, promotes lymphocyte proliferation and inhibits apoptosis. Immunoglobulin, a natural protein, neutralizes endotoxins and enhances the phagocytic ability of monocytes and macrophages. Its administration may benefit septic patients with multidrug-resistant bacterial infections. Thymosin alpha1 activates innate immune cells like dendritic cells (DCs), natural killer (NK) cells, and macrophages. This stimulation leads to T-cell proliferation and enhanced antibacterial effects of Th1 cells. Mesenchymal stem cells (MSCs) facilitate the maturation of M2 macrophages and regulatory T cells, promoting bacterial clearance and reducing excessive inflammation. This alleviates organ damage and ultimately reduces sepsis mortality. Coinhibitory molecule antibodies and antagonists targeting TIM-3, PD-1, BTLA, among others, can restore the function of both innate and adaptive immune cells, reversing the state of immune exhaustion. GM-CSF refers to granulocyte-macrophage colony-stimulating factor, MSC to mesenchymal stem cells, and BTLA to B and T lymphocyte attenuator. Created by biorender.com.

Sepsis is a complex condition often accompanied by complications such as ARDS, cardiac dysfunction, renal failure, and multi-organ failure, which significantly increase mortality and impact quality of life. Managing these complications is challenging due to the intricate pathogenesis involving multiple biological processes. However, recent research has made progress in sepsis treatment. Immunotherapy shows promise by modulating immune cells and cytokines to enhance immune function and improve antimicrobial and anti-inflammatory capabilities (**Figure 4**). Common strategies include using immune stimulants, immunomodulators, and activating specific immune cells to reduce inflammation and improve outcomes (57). However, challenges remain, including immune dysregulation complexity, individual variability, and optimizing treatment strategies. Further research and clinical practice are needed to develop more effective and personalized immunotherapy approaches to improve sepsis patient outcomes. The use of Toll-like receptors (TLRs) as therapeutic targets in sepsis treatment is an active area of research. Several approaches have been proposed, including developing TLR antagonists to inhibit signaling and reduce inflammation (58). For example, TLR4 antagonists have shown potential in preclinical studies. Another strategy is targeting downstream pathways like NF-κB signaling to attenuate inflammation. Immunomodulatory therapies, such as corticosteroids, have also been explored, but their use remains controversial. TLR2 and TLR4 are the most studied TLRs in sepsis, and selective targeting of these receptors using monoclonal antibodies has shown promise (59). However, the complex immune response in sepsis requires careful consideration (60). Further research is needed to assess the safety and efficacy of TLR-targeted therapies and to determine optimal timing for interventions. A comprehensive approach combining multiple strategies is needed to address the multifaceted immunopathology of sepsis (61).

The limitations of current research on Toll-like receptors (TLRs) in sepsis include the reliance on animal models, the lack of clinical validation, and the variability in TLR expression. Many findings in this area are primarily derived from animal studies, which may not fully recapitulate human physiology and pathology, limiting the direct translation of preclinical findings to clinical applications. Additionally, there is a scarcity of clinical trials that validate the therapeutic potential of targeting TLRs in sepsis and its complications, highlighting the need for robust clinical data to support the efficacy and safety of such interventions in human patients. Furthermore, the diversity in TLR expression levels and functions among individuals and tissues poses a challenge in interpreting research findings and applying them to heterogeneous patient populations, necessitating a more nuanced understanding of TLR biology in different clinical contexts. Moving forward, key issues for future research on TLRs in sepsis involve conducting mechanistic studies, human-specific investigations, and therapeutic targeting strategies. Mechanistic studies are essential to unravel the precise molecular mechanisms through which TLRs contribute to organ damage in sepsis, including their crosstalk with other signaling pathways and downstream effectors (62). Moreover, there is a critical need for human studies that evaluate TLR function and regulation in sepsis patients to delineate the clinical relevance of TLR-mediated responses in disease progression and outcomes. Lastly, future research efforts should focus on developing and evaluating novel therapeutic interventions that target TLR signaling pathways, with a specific emphasis on ensuring the safety and efficacy of these approaches in clinical settings, thereby paving the way for potential translation into clinical practice. Addressing these key issues will advance our understanding of TLRs in sepsis pathophysiology and facilitate the development of novel therapeutic strategies for improving patient outcomes.
